# Recurrent Urosepsis Following Stent Removal for Ureteral Stones: A Case Report

**DOI:** 10.1155/criu/5547651

**Published:** 2025-04-23

**Authors:** Cesar Prugue, Parker Reber, Amanda Austin

**Affiliations:** ^1^Edward Via College of Osteopathic Medicine, Spartanburg, South Carolina, USA; ^2^Gateway Family Medicine, Bon Secours, Travelers Rest, South Carolina, USA

## Abstract

Urosepsis, a severe infection originating from the urinary tract, can be life-threatening. We present the case of a 56-year-old female who developed urosepsis twice within 15 days, each episode occurring shortly after stent removal. Ureteroscopy with stent placement was initially performed to treat a 12-mm stone in the distal left ureter. Although the stent was removed only after imaging showed no residual stones, sepsis developed shortly after, leading to another ureteroscopy and stent placement. Before the removal of the second stent, imaging again confirmed no stones were present, yet she experienced sepsis once more following the second stent removal. Further imaging studies during hospital admission for both episodes of sepsis revealed stone fragments and hydronephrosis which were missed during office evaluations. This case highlights the need for more effective imaging techniques to detect residual stones. The decision to place a stent after ureteroscopy for ureteral stone treatment should also be carefully considered, even for low-risk patients, to reduce infection risk.

## 1. Introduction

Urosepsis is a serious infection that originates in the urinary tract and can lead to high mortality if not treated promptly [1]. The risk of developing urosepsis after ureteroscopic procedures for kidney stones is around 5.0% [2]. Patients with conditions such as older age, diabetes, and heart disease have a higher risk of developing urosepsis after ureteroscopic interventions. Double J stents are commonly placed after ureteroscopy (URS) to prevent blockages caused by stone fragments, but stent use varies widely and they are routinely placed even when omission criteria are met [3, 4]. More than 1.5 million ureteral stents are used in the world each year [5].

We present the case of a 56-year-old female with a past medical history of kidney stones and hyperlipidemia who developed urosepsis twice within 15 days, each time following stent removal. After the first stent was removed, she developed urosepsis and required placement of another stent. Shortly after the removal of the second stent, she again experienced urosepsis. This recurrence raises concerns about both the necessity of stent placement in low-risk patients and the adequacy of current imaging techniques in detecting residual stone fragments. It highlights the need for improved imaging strategies and a reevaluation of stent placement protocols to prevent complications like urosepsis.

## 2. Case Presentation

A 56-year-old female with a history of right kidney stones and hyperlipidemia was noted to have a mildly elevated alkaline phosphatase level of 183 IU/L during her annual wellness visit. Follow-up lab work 1 month later showed a decrease in alkaline phosphatase level to 159 IU/L, which was still slightly higher than normal. Her primary care physician (PCP) initially attributed this to possible fatty liver associated with hyperlipidemia and recommended dietary modifications. Over the next few months, as the alkaline phosphatase levels remained stable but still slightly elevated, an abdominal ultrasound was ordered to assess the liver and bile ducts.

**Figure 1:**
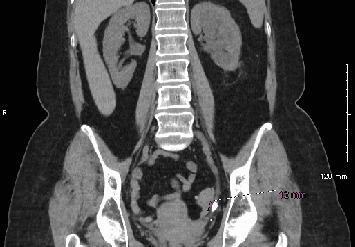
Coronal section showing moderate left hydronephrosis secondary to a 12-mm stone in the distal left ureter.

The ultrasound revealed a widened bile duct and incidentally detected mild to moderate left-sided hydronephrosis. There were no kidney stones noted on the ultrasound. Concerned about possible obstruction, CT scans of the abdomen and pelvis were ordered. Imaging confirmed a 12-mm stone in the distal left ureter, causing moderate hydronephrosis and hydroureter (Figure 1).

**Figure 2:**
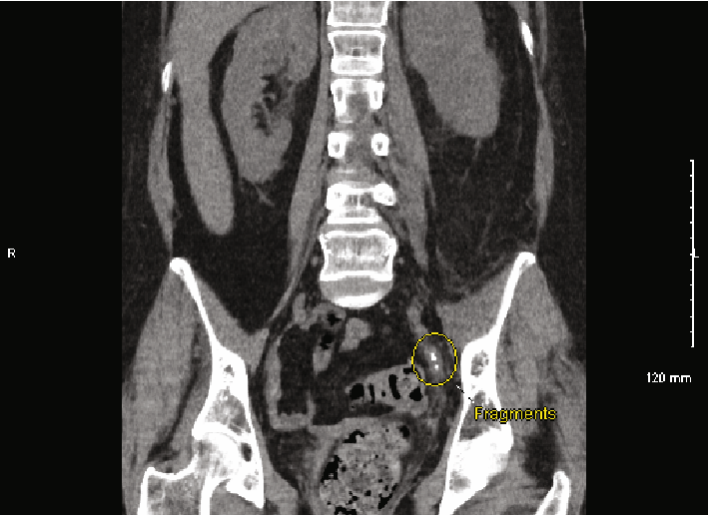
Moderate hydroureteronephrosis and left perinephric stranding due to multiple stones measuring up to 3 mm in width within the distal left ureter.

The patient was referred to a urologist, who performed a left URS with laser lithotripsy and stent placement to address the obstruction. One week later, kidney, ureter, and bladder (KUB) imaging did not reveal any residual stones, and the stent was removed. However, just 3 days later, she presented to the emergency department (ED) with fever, chills, and left flank pain. Her white blood cell (WBC) count was 19.2 K/*μ*L, lactate was 1.0 mmol/L, and procalcitonin was 5.36 ng/mL. CT imaging demonstrated moderate hydroureteronephrosis with residual stone fragments (Figure 2).

Blood cultures were positive for *Escherichia coli*, and she was diagnosed with sepsis secondary to a urinary tract infection (UTI) and obstructive pyelonephritis. She was treated with intravenous ceftriaxone and underwent another URS with stent placement.

**Figure 3:**
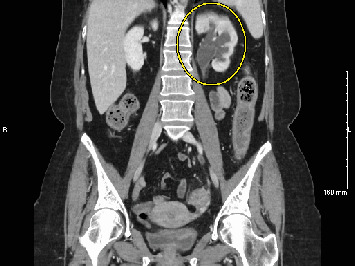
Moderate to severe left-sided hydroureteronephrosis with interval decrease in stone burden.

Twelve days after the repeat URS with stent placement, she was seen for stent removal. KUB imaging showed the left ureteral stent in the correct position and no visible stones. The stent was removed, and the patient was prescribed Vantin (cefpodoxime) by her urologist, but 3 days later she presented to her PCP with chills, nausea, vomiting, and diarrhea. Due to high suspicion of infection, blood tests were ordered which revealed a WBC of 29.6 K/*μ*L. She was advised to return to the ED, where she presented with fever, tachycardia, and an elevated WBC count of 24.7 K/*μ*L. Lactate was 2.7 mmol/L, and procalcitonin was 1.08 ng/mL. CT imaging showed moderate to severe left-sided hydroureteronephrosis and a residual 2-mm stone in the distal ureter (Figure 3).

She was treated empirically for sepsis with intravenous antibiotics and supportive care.

## 3. Discussion

There are few published cases of recurrent urosepsis in patients, several of which involve elderly individuals with significant comorbidities. In fact, the average age of a patient with sepsis is around 65 [6]. For example, Khoo et al. described the case of a 77-year-old woman with recurrent urosepsis in addition to complications from pheochromocytoma, cardiomyopathy, and a fatal myocardial infarction [7]. Another case of recurrent urosepsis published by Krueger et al. included a 25-year-old woman; however, this patient was complicated by chronic reflux into the renal pelvis due to neurogenic bladder impairment caused by lumbar meningomyelocele [8].

According to the American Urological Association, uncomplicated ureteral stones ≤ 10 mm can be managed without intervention, while larger stones may require URS [9]. The decision to not place a stent after URS is based on specific criteria, such as the absence of ureteral strictures or other anatomical factors that may prevent stone clearance. Although stent placement is common, its necessity, particularly in low-risk patients, remains uncertain. Recent studies suggest that stenting may not significantly reduce complications such as UTIs or the need for secondary interventions [10]. While it might slightly reduce unplanned return visits, the benefit is unclear. These findings support an individualized approach to stent use, where omission in low-risk patients may be considered, as the risks, such as postoperative pain, may outweigh the benefits. Given the potential for stent placement to increase the risk of sepsis, further investigation is required [11]. Additionally, the use of prophylactic antibiotics after URS to prevent infection is also an area that needs more research [12].

Stent removal usually occurs between 5 and 7 days after successful URS and is usually preceded by imaging such as ultrasound or KUB to ensure there are no residual stone fragments [13]. In this case, KUB was done in the clinic before stent removal on both occasions but failed to detect remaining stone fragments that were later found on subsequent CT scans. The use of ultrasound and KUB is reasonable for reducing radiation exposure; however, CT imaging is the most accurate for identifying kidney stones [14]. Improved imaging modalities prior to stent removal are crucial to ensure complete stone clearance. Low-dose (LD) and ultralow-dose (ULD) CT protocols, which maintain high diagnostic accuracy while reducing radiation exposure, represent a promising alternative for detecting residual stones [15]. Without an accurate imaging modality, remaining fragments can lead to infection and ultimately urosepsis, as demonstrated in this case.

## 4. Conclusion

A middle-aged female without significant medical history experienced recurrent episodes of urosepsis within a short time frame following stent removal. This case highlights the potential complications of URS and instrumentation, including stent placement and removal, in the management of ureteral stones. Future studies should focus on refining imaging and stent management practices to prevent recurrent complications like urosepsis in patients undergoing URS for ureteral stones.

## Data Availability

No datasets were generated or analyzed during the preparation of this case report.
